# 398. Post-Infectious Irritable Bowel Syndrome in Patients Referred to a Complicated *Clostridioides difficile* Clinic

**DOI:** 10.1093/ofid/ofac492.476

**Published:** 2022-12-15

**Authors:** Natalie Pham, Marieke Jones, Deiziane Costa, Jae Shin, Brian Behm, Cirle A Warren

**Affiliations:** University of Virginia, Charlottesville, Virginia; University of Virginia, Charlottesville, Virginia; University of Virginia, Charlottesville, Virginia; University of Virginia, Charlottesville, Virginia; University of Virginia, Charlottesville, Virginia; University of Virginia, Charlottesville, Virginia

## Abstract

**Background:**

Recurrence of *Clostridioides difficile* infection (CDI) occurs in up to 20-30% after an initial episode and up to more than half of patients with previous infection. *C. difficile* has also been associated with post-infectious irritable bowel syndrome (PI-IBS) following the resolution of an acute infection, complicating assessment of gastrointestinal symptoms post-CDI. The prevalence and risk factors for post-CDI IBS are not as well-defined as PI-IBS secondary to other pathogens.

**Methods:**

In this study, we examined the electronic medical record of 67 consecutive patients referred to a Complicated *C. Difficile* Clinic from March 2020 to July 2021 for consideration of fecal microbiota transplantation.

**Results:**

Patients were referred by primary care providers (54%), gastroenterologists (16%), hospital admitting team (19%) or others (10%). Mean age was 64 (21-93) and 51 patients (76%) were female. In 25 out of the 67 patients (37%), treatment was prescribed without documentation of a positive *C. difficile* assay. We found that 32.8% of patients referred to the clinic for recurrent CDI had symptoms consistent with post-CDI IBS. Patients with post-CDI IBS were generally younger in age (P = 0.03), had fewer medical comorbidities (P = < 0.001), and more often had history of anxiety (P = 0.03) than patients with a diagnosis of CDI.

**Conclusion:**

Our findings suggest that post-CDI IBS is prevalent, can be misdiagnosed as CDI recurrence, and may lead to inappropriate use of anti-*C. difficile* agents or interventions.

**Disclosures:**

**All Authors**: No reported disclosures.

